# Editorial: Volatilomics in plant and agricultural research: recent trends

**DOI:** 10.3389/fpls.2023.1289998

**Published:** 2023-09-29

**Authors:** Deepak Kasote, Jisun Lee, Nese Sreenivasulu

**Affiliations:** ^1^ Plants for Human Health Institute, North Carolina State University, Kannapolis, NC, United States; ^2^ Department of Plant Science and Technology, Chung-Ang University, Anseong, Republic of Korea; ^3^ Rice Breeding Innovation Department, International Rice Research Institute, Los Baños, Philippines

**Keywords:** volatilomics, plant-plant communication, pathogen, cultivars, GC-MS

Plants synthesize volatile organic compounds (VOCs) that function as signaling molecules. These VOCs are essential in plant defense against herbivores and insects and play a vital role in plant interaction with biotic and abiotic elements in the environment. Moreover, the composition of VOCs of consumable fruits and grains defines aroma and flavor, thereby considerably deciding consumers’ preferences towards them. In general, the set of biosynthesized volatiles is called volatilome, and analysis of such volatilomes using gas chromatography-mass spectrometry (GC-MS)-based targeted and untargeted approaches is usually referred to as volatilomics. The field of volatilomics is continuously growing and evolving with rapid development in analytical and data processing methods. Hence, capturing recent advances and applications in volatilomics is becoming valuable in advancing interdisciplinary research in plant sciences. The goal of this Research Topic was to collect recent research highlights about the application of volatilomics, both targeted and untargeted in different research areas of plant and agricultural sciences. Under this Research Topic, seven research articles are published that highlight the recent applications of GC-MS-based volatilomics in screening breeding lines/cultivars, understanding the effect of growing conditions on aroma-based fruit quality to optimize quality preferences and unraveling volatiles-based plant-plant communication, including the potential of volatilomics in identifying novel antibacterial and insect repellent volatile compounds.

## Volatilomics to screen breeding lines/cultivars

Rapid screening of breeding lines/cultivars is crucial for accelerating breeding programs, including efficient selection of genotypes with desired quality traits such as aroma. Volatilomics is found to be valuable in the rapid characterization of genotypes based on the composition of VOCs. Jo et al. used a GC-MS-based untargeted volatilomics approach to differentiate 20 representative cucumber lines from various geographical locations such as Korea, Europe, and Thailand. In this study, authors demonstrated that the analysis of volatiles from the flesh of cucumber breeding lines provides a more distinct metabolite pattern than that obtained from the peel of these lines. In addition, it was shown that key cucumber VOCs such as 2-hexenal, 2,4-nonadienal, and 2,6-nonadienal are relatively low in Korean cucumber lines which makes them less flavor intense. Similarly, Deng et al. used an optimized GC-MS headspace method for leaf volatilome fingerprinting of 42 citrus cultivars and showed the utility of the volatilomics tool in citrus species identification, including chemotaxonomic studies. The key VOCs that contributed to distinguishing these citrus cultivars include thymol derivatives (cis-sabinene hydrate, sabinene, thymol, and thymol methyl ether), which are usually involved in defense response. Moreover, it was reported that (Z)-3-hexen-1-ol contributing to green aroma had a role in distinguishing orange and mandarian groups of citrus cultivars. Quan et al. employed two-dimensional gas chromatography quadrupole time of flight mass spectrometry (GC×GC-QTOF-MS) to investigate the volatilomes of four cultivars of Chinese rose. Analysis of a total of 122 volatiles showed significant quantitative variation of VOCs among the different cultivars. Rosa Crimson Glory contained high concentrations of phenyl acetate, rose oxide, trans-rose oxide, phenylethyl alcohol, and 1,3,5-trimethoxybenzene, which are all described as flowery and rose-like descriptors. Ros Blue River and Rosa Funkuhr both had significant amounts of 3,5-dimethoxytoluene and phenylethyl alcohol, respectively.

## Volatilomics to understand the effect of growing conditions on aroma-based fruit quality

Aroma-active VOCs are used in defining fruit quality. For example, in strawberries, furanones contribute to a sweet aroma that increases during ripening, while aldehydes such as hexanal and (*E*)-2-hexanal contribute to a fresh aroma, which decreases during ripening. Although the aroma is genotype-dependent, the perceived aroma of agricultural products is strongly affected by growing and environmental conditions (Mostafa et al., 2022). Cho et al. studied the volatilome of strawberries as a shelf-life indicator to understand the application of drainage ratios (controlled irrigation and means of drainage) in improving fruit quality. The authors demonstrated that drainage ratios could improve fruit quality by minimizing the accumulation of VOCs linked to over-ripening such as hexanal and ethyl hexanoate.

## Volatilomics to unravel the effect of weed volatiles on plant growth, yield, and quality

It is known that VOCs mediate plant-plant as well as plant-pathogen and -herbivore interactions. Research interest in understanding the role of VOCs in plant-plant “communication” and the perception of volatiles has increased substantially (Bouwmeester et al., 2019). Sakurai et al. examined the effect of VOCs exposure from artificially damaged weeds (mugwort and tall goldenrod, individual and mixture) on maize growth and reproduction. The study showed that defensive responses in maize leaves against herbivores such as common armyworms were induced due to the exposure to weed volatiles. Moreover, maize seedlings exposed to goldenrod-specific or mixed volatiles produced more leaves and tillers than control plants. Interestingly, maize seedlings treated with volatiles generated from the damaged weeds increased the number of female ears and sugar content in kernels. The study also observed an elevated salicylic acid content in seedlings upon volatiles exposure.

## Volatilomics to identify novel antibacterial and insect repellents

Plant essential oils that contain complex mixtures of low-molecular-weight, highly volatile compounds have been proven to be promising biopesticides (Devrnja et al., 2022). Analysis and characterization of volatiles in essential oils is a crucial part of studying promising novel antibacterial and insect repellents. Patel et al. demonstrated antibacterial (against pathogens of *Pseudomonas cichorii*, *P. syringae* and *Xanthomonas perforans*) and insect (bed bug) repellent activities of essential oils from novel cultivars of catnip (*Nepeta cataria* L. cv. CR9) and oregano (*Origanum vulgare* L. cv. Pierre) that are rich in the terpenes nepetalactone and carvacrol, respectively. Similarly, Gomes et al. studied the effect of successive harvesting on the essential oil composition of catnip cultivars using GC-MS. It was found that agronomic practices can significantly affect the accumulation of VOCs, mainly arthropod-repellent iridoid terpenes, in catnip genotypes. In a nutshell, these articles highlight the significance of volatilomics in developing novel plant-based antibacterial and insect repellents for agricultural pest and pathogen management.

## Author contributions

DK: Writing – original draft, Writing – review & editing. JL: Writing – review & editing. NS: Writing – review & editing.
